# Intervention or Conservative Management for Symptomatic Cerebral Cavernous Malformations: A Prospective, Population-Based Cohort Study

**DOI:** 10.1212/WNL.0000000000218442

**Published:** 2026-08-12

**Authors:** Abel Clemens Adriaan Sandmann, Dagmar Verbaan, Jonathan M. Coutinho, William Peter Vandertop, Philip Michael White, Rustam Al-Shahi Salman

**Affiliations:** 1 Department of Neurology, Amsterdam UMC, University of Amsterdam, the Netherlands;; 2 Amsterdam Neuroscience, Neurovascular Disorders, the Netherlands;; 3 Department of Neurosurgery, Amsterdam UMC, University of Amsterdam, the Netherlands;; 4 Translational and Clinical Research Institute, Newcastle University, United Kingdom; and; 5 Institute for Neuroscience and Cardiovascular Research, The University of Edinburgh, United Kingdom.

## Abstract

**Background and Objectives:**

There is ongoing clinical equipoise regarding the optimal treatment strategy for symptomatic cerebral cavernous malformations (CCMs). The aim of this study was to estimate the causal effect between CCM intervention (microsurgical resection or stereotactic radiosurgery) and clinical outcomes during long-term follow-up.

**Methods:**

This population-based cohort study included patients (aged 16 years or older) newly diagnosed with CCM using brain MRI or pathology in Scotland during 1999–2003 or 2006–2010. Using Cox regression analysis, we evaluated associations between CCM intervention (as a time-dependent covariate) and a primary outcome of persistent functional impairment or death (defined as at least 2 consecutive annual Oxford Handicap Scale [OHS] scores of ≥2, with sensitivity analysis of OHS scores ≥3), a composite of symptomatic intracranial hemorrhage or new persistent/progressive nonhemorrhagic focal neurologic deficit (ICH/FND) definitely/possibly related to CCM or CCM intervention, and epileptic seizure(s) definitely/possibly related to CCM during prospective follow-up.

**Results:**

Among 306 patients with CCM, 190 were symptomatic (89 [47%] because of ICH/FND and 101 [53%] because of epileptic seizure(s), median age 40 years [interquartile range (IQR) 31–55], and 95 [50%] women). During a median follow-up of 16 years (IQR 14–21, completeness 95%), 37 (19%) patients underwent CCM intervention (n = 35 microsurgical resection and n = 2 stereotactic radiosurgery); they were younger (median 33 [26–40] vs 43 [32–59] years, *p* < 0.001), had presented with ICH more often (18/37 [49%] vs 35/153 [23%], *p* = 0.002), and had brainstem CCM less frequently (3/37 [8%] vs 36/153 [24%], *p* = 0.037) than those managed conservatively. CCM intervention was not associated with persistent functional impairment or death when analyzing OHS scores of ≥2 but was associated with OHS scores ≥3 (adjusted hazard ratio [HR] 2.66 [95% CI 1.19–5.94], *p* = 0.017). CCM intervention was associated with ICH/FND definitely related to CCM or CCM intervention during follow-up (adjusted HR 3.59 [95% CI 1.05–12.20], *p* = 0.041). CCM intervention was not associated with epileptic seizure(s) during follow-up (adjusted HR 1.56 [95% CI 0.49–4.99], *p* = 0.45).

**Discussion:**

CCM intervention for symptomatic CCM was associated with persistent functional dependence or death during long-term follow-up. This may be explained by ICH/FND definitely related to CCM or CCM intervention. These findings should be investigated in a definitive randomized controlled trial.

## Introduction

Cerebral cavernous malformations (CCMs) are intracranial vascular malformations that are generally sporadic and solitary, although 1 in 5 cases have multiple lesions, often related to a familial form.^1^ Diagnosis of CCM may occur after the onset of epileptic seizures or focal neurologic deficits (FND) anatomically attributable to CCM, either of which may be provoked by intracranial hemorrhage (ICH).^2^ CCM may also be an incidental discovery if brain imaging is performed in asymptomatic people or those with unrelated symptoms. A CCM diagnosis can significantly reduce patients' quality of life^3^ and may prompt consideration of CCM intervention (i.e., microsurgical resection or stereotactic radiosurgery [SRS]).

There is ongoing debate regarding the optimal treatment strategy for CCM. Although incidental CCMs are usually managed conservatively given their benign clinical course,^4^ there is clinical equipoise about the optimal treatment of symptomatic CCM, whether it may be intervention or conservative management. A pilot phase randomized controlled trial (RCT) did not show a difference between these strategies but indicated that a definitive RCT might be feasible.^5^ In current clinical practice, physicians and patients weigh the estimated up-front risks of CCM intervention against studies of the untreated clinical course.^6,7^ However, such studies allow only indirect comparisons and censor patients at intervention without capturing outcomes resulting from interventions.

Nonrandomized comparative studies can provide some guidance for treatment decisions in CCM, provided that selection bias and confounding are addressed. However, such studies remain scarce, a limitation consistently reported as a gap in the literature.^8^ A propensity score–matched analysis of 34 pairs found no significant difference between microsurgical resection and conservative management of CCM, but this study had a retrospective, hospital-based design with limited follow-up.^9^ A previous report of the first cohort in our prospective, population-based study had longer follow-up, but may have been affected by immortal time bias.^10^ The objective of the current study was to analyze symptomatic CCM in the entire cohort, estimating the causal effect between CCM intervention and clinical outcomes during long-term follow-up,^11^ to test the hypothesis that conservative management is superior to CCM intervention.^10^

## Methods

### Study Design

In this prospective, population-based cohort study, patients with CCM were identified by the Scottish Audit of Intracranial Vascular Malformations (SAIVMs).^12^ SAIVMs was a National Health Service clinical audit and included patients aged 16 years or older who had a first-ever diagnosis using brain MRI or pathology of any type of intracranial vascular malformation during 1999–2003 or 2006–2010, while being permanent residents of Scotland. Multiple overlapping sources of case ascertainment were used, which involved a Scotland-wide collaborative network of specialists in neurology, neurosurgery, stroke medicine, radiology, and pathology, as well as centralized, routine coding of hospital discharge records and death certificates.

### Standard Protocol Approvals, Registrations, and Patient Consents

This study of solely anonymized extracts was approved by the Multicenter Research Ethics Committee for Scotland (MREC/98/0/48) and the Fife and Forth Valley Research Ethics Committee (08/S0501/76). The observational studies (collection and analysis of routinely coded clinical data without any changes to care) required opt-out consent, whereas the annual postal questionnaire studies of self-reported data required written informed consent at enrolment. The audit and research protocols were published on the SAIVMs website and with the Directory of Clinical Databases and mention the prespecified objective “to monitor the outcomes of patients who do and do not receive interventional treatment (and thereby monitor the beneficial and adverse effects of these interventions).”

### Data Collection

The inception point of this study was the date of first onset of symptoms at initial CCM symptomatic presentation or, if the CCM was an incidental discovery, during follow-up. Certified neuroradiologists used brain images to verify each CCM diagnosis according to established criteria,^13^ examine radiographic evidence of recent ICH, and collect several CCM characteristics: number of CCMs, location of CCMs (deep or superficial), presence of a brainstem CCM, temporal cortical/superficial CCMs or associated developmental venous anomaly, and the largest diameter of CCM in any direction. Superficial CCMs were defined as those involving the cortex or pial surface, whereas deep CCMs were located in the remaining areas. In case of multiple CCMs, we analyzed the treated CCM or, if managed conservatively, the symptomatic CCM. The presence of a brainstem CCM and/or temporal cortical/superficial CCM was noted separately to capture all patients with CCM in these locations. Baseline characteristics were collected from hospital medical records and included demographics, comorbidities, and medicines. Data on race or ethnicity were not available for all patients in SAIVMs because they were not routinely collected. Baseline functional status was assessed on the Oxford Handicap Scale (OHS), which is a variant of the modified Rankin Scale, ranging from 0 (no handicap) to 6 (death).^14^

During follow-up, occurrence of clinical outcome events, CCM interventions, and functional status on the OHS was assessed prospectively until December 31, 2023, using uninterrupted annual surveillance of primary care practitioners' and hospitals' medical records as well as annual postal questionnaires to primary care practitioners and consenting participants on each anniversary after the CCM diagnosis. Because OHS scores were assessed annually, the inception OHS score in patients who were symptomatic for the first time during follow-up (after an initial incidental diagnosis) was the earliest annual rating recorded after that event. Furthermore, treatment decisions during follow-up were left to the discretion of a patient's physician.

Outcome events were adjudicated using hospital medical records, brain images, and pathology reports. Events were classified as ICH if the symptoms were anatomically consistent with timely radiologic, pathologic, surgical, or CSF investigation evidence of a recent hemorrhage or as FND or epileptic seizure(s) if there was no such evidence.^2^ Only persistent or progressive FND was analyzed, excluding transient symptoms (lasting <24 hours). All events were classified as definitely, possibly, or not related to CCM or attributable to CCM intervention. Events were considered unrelated to CCM if the clinical features were not anatomically referable to CCM or if another cause was identified. Events possibly related to CCM were distinguished from events that were definitely related to CCM if another cause (e.g., ischemic stroke) was possible and brain imaging neither identified a CCM-related cause nor confirmed an alternative cause. In case of death, we determined its cause by reviewing death certificates, medical records, and autopsy reports (if a postmortem had been conducted).

### Outcomes of Interest

The primary outcome was persistent functional impairment or death, which was defined as at least 2 consecutive annual OHS score ratings of ≥2 (signifying “minor handicap; some restriction to lifestyle, but able to look after themself,” or worse). We performed sensitivity analysis of OHS score ratings of ≥3 (signifying “moderate handicap; significant restriction to lifestyle, and requires some assistance” or worse). We only used OHS score ratings obtained after the inception OHS score to determine progression to this outcome. Because OHS scores were rated annually, the exact timing of these outcomes was not directly observed. We therefore defined the event time as the midpoint between inception or the most recent OHS scores 0–1 (or 0–2) and the first of the successive OHS score rating ≥2 (or ≥3). In case of a death, we selected the date of death. If a rating was missing, we assumed that the same OHS score persisted until the subsequent rating. To facilitate interpretation from an interventional perspective, we used the same approach to also assess the reciprocal sustained favorable functional outcome, defined as at least 2 consecutive annual OHS score ratings of 0–1, with sensitivity analysis of OHS scores of 0–2.

The secondary outcomes included a composite outcome of symptomatic ICH or new nonhemorrhagic persistent/progressive FND (ICH/FND) during follow-up as well as epileptic seizure(s) during follow-up. We used a composite outcome of ICH/FND because the severity of these events seemed similar,^15^ and some new FND events might be undetected ICH.^2^ To facilitate comparison with studies focusing exclusively on ICH, we also analyzed symptomatic ICH alone. Furthermore, we quantified death related to CCMs. We primarily analyzed events that were definitely or possibly related to CCMs (or attributable to CCM intervention, if applicable) and performed sensitivity analysis of events that were only definitely related to CCMs (or attributable to CCM intervention, if applicable).

### Statistical Analysis

At the time of inception, we performed between-group comparisons between patients undergoing CCM intervention during follow-up and those managed conservatively throughout, using the Mann-Whitney *U* test for continuous variables and the χ^2^ test (or the Fisher exact test if expected cell counts were less than 5) for categorical variables. We calculated completeness of prospective follow-up data by dividing the total observed follow-up time by the total potential follow-up time before death or the final follow-up date of SAIVMs (December 31, 2023).^16^ We censored follow-up at the date of death or last available follow-up, whichever occurred earlier.

The primary exposure was CCM intervention. Because treatment allocation was not randomized, indicating lack of causal inference, we performed an association study, estimating the causal effect between the primary exposure and outcomes of interest.^11^ We performed multiple Cox proportional hazards regression analyses, with at least 7 events per covariate in each model.^17^ Because CCM intervention may be performed at any time after diagnosis and treatment status can change during follow-up, we included the primary exposure as a time-dependent covariate. This ensures that events before CCM intervention are attributed to untreated exposure time and events after CCM intervention are attributed to treated exposure time. Additional covariates were not time dependent. We calculated unadjusted and adjusted hazard ratios (HRs) with corresponding 95% CIs and used log-log curves to check the proportional hazards assumption. We used Cox models to plot unadjusted survival curves for each outcome of interest, with estimated exposure times in the untreated and treated states, calculating standard errors clustered at the patient level to account for multiple observation intervals per patient.

In addition to treatment status, each Cox regression analysis also included the demographic factors age and sex. Potential confounders of the association between the primary exposure and outcomes of interest were added as additional covariates based on directed acyclic graphs (eFigures 1–3). For functional outcome, we sequentially considered the following for inclusion: Charlson Comorbidity Index (CCI) score, social deprivation category, type of symptoms at inception (ICH/FND or epileptic seizure(s)), inception OHS score, presence (vs absence) of brainstem CCMs, multiple (vs single) CCMs, and largest CCM diameter. Each patient received a CCI score (ranging 0–33) based on their comorbidities,^18^ using age as a separate covariate. Deprivation category is a composite measure reflecting the socioeconomic status of a geographical area in Scotland, based on income, employment, education, housing, health, and access to services, ranging from 1 (most deprived) to 7 (least deprived).^19^ Patients were assigned a deprivation score according to their area of residence at the initial presentation.

Additional covariates sequentially considered for inclusion in the model for ICH/FND during follow-up were ICH/FND (vs epileptic seizure[s]) at inception, presence (vs absence) of brainstem CCM, antithrombotic (i.e., antiplatelet or anticoagulant) medication at inception, multiple (vs single) CCMs, and largest CCM diameter. For epileptic seizure(s) during follow-up, we sequentially considered epileptic seizure(s) (vs ICH/FND) at inception, presence (vs absence) of temporal cortical/superficial CCMs, antiseizure medication after inception, multiple (vs single) CCMs, and largest CCM diameter. Analyses were performed using IBM SPSS Statistics version 28^20^ and RStudio version 2024.12.1.^21^

### Data Availability

Deidentified participant data supporting the findings reported in this study will be made available for scientific research upon reasonable request to the corresponding author and consequent approval of the proposal.

## Results

### Inception and Follow-Up

From 1999 to 2003 and 2006 to 2010, a total of 306 adult residents in Scotland had a first-in-a-lifetime diagnosis of at least 1 CCM (295 on brain MRI, 6 at autopsy, and 5 after surgical excision). We excluded 116 cases who were diagnosed incidentally and did not experience any symptoms due to CCMs during follow-up, of whom 7 underwent CCM intervention (details are presented in eTable 1). Among 190 included patients with symptomatic CCM, 21 (11%) experienced CCM-related symptoms for the first time during follow-up, a median of 2 years (interquartile range [IQR] 1–8) after an incidental diagnosis, and 169 were symptomatic at diagnosis. At inception, the median age was 40 years (IQR 31–55) and 95 (50%) patients were women (Table 1). Types of symptoms at inception were ICH/FND in 89 (47%) patients and epileptic seizure(s) in 101 (53%) patients. Two patients who had initially presented incidentally had an inception OHS score of 6. One died on the day of an ICH definitely related to CCMs. The other died 8 days after an ICH possibly related to CCM, but the cause of death recorded on the death certificate was ischemic stroke.

**Table 1:** Baseline Characteristics of Patients With Symptomatic CCM

Variable	All patients (n = 190)	CCM intervention (n = 37)	Conservative management (n = 153)	*p* Value
Age (y)	40 (31–55)	33 (26–40)	43 (32–59)	**<0.001**
Sex (female)	95 (50)	19 (51)	76 (50)	0.86
CCI score				
0	144 (76)	33 (89)	111 (73)	**0.042**
1	17 (9)	1 (3)	16 (10)	
2	14 (7)	1 (3)	13 (8)	
≥3	15 (8)	2 (5)	13 (8)	
Deprivation category				
1–2 (most deprived)	50 (26)	7 (19)	43 (28)	0.80
3	50 (26)	12 (32)	38 (25)	
4	44 (23)	11 (30)	33 (22)	
5–7 (least deprived)	46 (24)	7 (19)	39 (25)	
First symptomatic event				
ICH/FND	89 (47)	20 (54)	69 (45)	0.33
ICH	53 (28)	18 (49)	35 (23)	**0.002**
FND	36 (19)	2 (5)	34 (22)	**0.019**
Epileptic seizure(s)	101 (53)	17 (46)	84 (55)	0.33
Multiple CCMs	43 (23)	10 (27)	33 (22)	0.48
Location of CCMs				
Deep	96 (51)	12 (32)	84 (55)	**0.014**
Superficial	94 (49)	25 (68)	69 (45)	
Brainstem CCM	39 (21)	3 (8)	36 (24)	**0.037**
Temporal cortical/superficial CCM	44 (23)	9 (24)	35 (23)	0.85
Largest CCM diameter (mm)	13 (9–18)	17 (8–20)	12 (10–18)	0.19
Associated DVA	13 (7)	5 (14)	8 (5)	0.14
OHS score^a^				
0	0 (0)	0 (0)	0 (0)	0.32
1	49 (26)	8 (22)	41 (27)	
2	121 (64)	23 (62)	98 (64)	
3	14 (7)	6 (16)	8 (5)	
4	3 (2)	0 (0)	3 (2)	
5	1 (1)	0 (0)	1 (1)	
6	2 (1)	0 (0)	2 (1)	

Abbreviations: CCI = Charlson Comorbidity Index; CCM = cerebral cavernous malformation; DVA = developmental venous anomaly; FND = nonhemorrhagic focal neurologic deficit; ICH = symptomatic intracranial hemorrhage; IQR = interquartile range; OHS = Oxford Handicap Scale.

Data are represented as median (IQR) or n (%); *p* values presented in bold were considered statistically significant (*p* < 0.05).

a At the first symptomatic event or, if that occurred during follow-up, the first OHS score thereafter.

Patients were followed for 2,989 person-years (median 16 years [IQR 14–21, range 0–25]) of 3,145 potential person-years of follow-up, resulting in an overall completeness of follow-up of 95%. During follow-up, 37 (19%) patients underwent CCM intervention (n = 35 microsurgical resection [all complete excisions, n = 1 twice for 2 CCMs], n = 2 SRS), a median of 10 months (IQR 3–17, range 0–8 years) after the first symptoms attributable to CCM. At inception, patients who underwent CCM intervention during follow-up were younger, had fewer comorbidities, presented more often with ICH and less often with FND, and harbored less frequently deep CCMs or brainstem CCMs than those managed conservatively throughout (Table 1). During follow-up, 35 (18%) patients died, of whom 29 had been managed conservatively and 6 had undergone CCM intervention. Seven deaths were definitely related to CCMs, including 2 in patients who had previously undergone CCM intervention (2 and 13 years later) and 1 death was possibly related to CCMs (details on causes of death are presented in eTable 2). Details on the outcomes of patients who underwent SRS are presented in eTable 3.

### Functional Outcome

**Figure 1:**
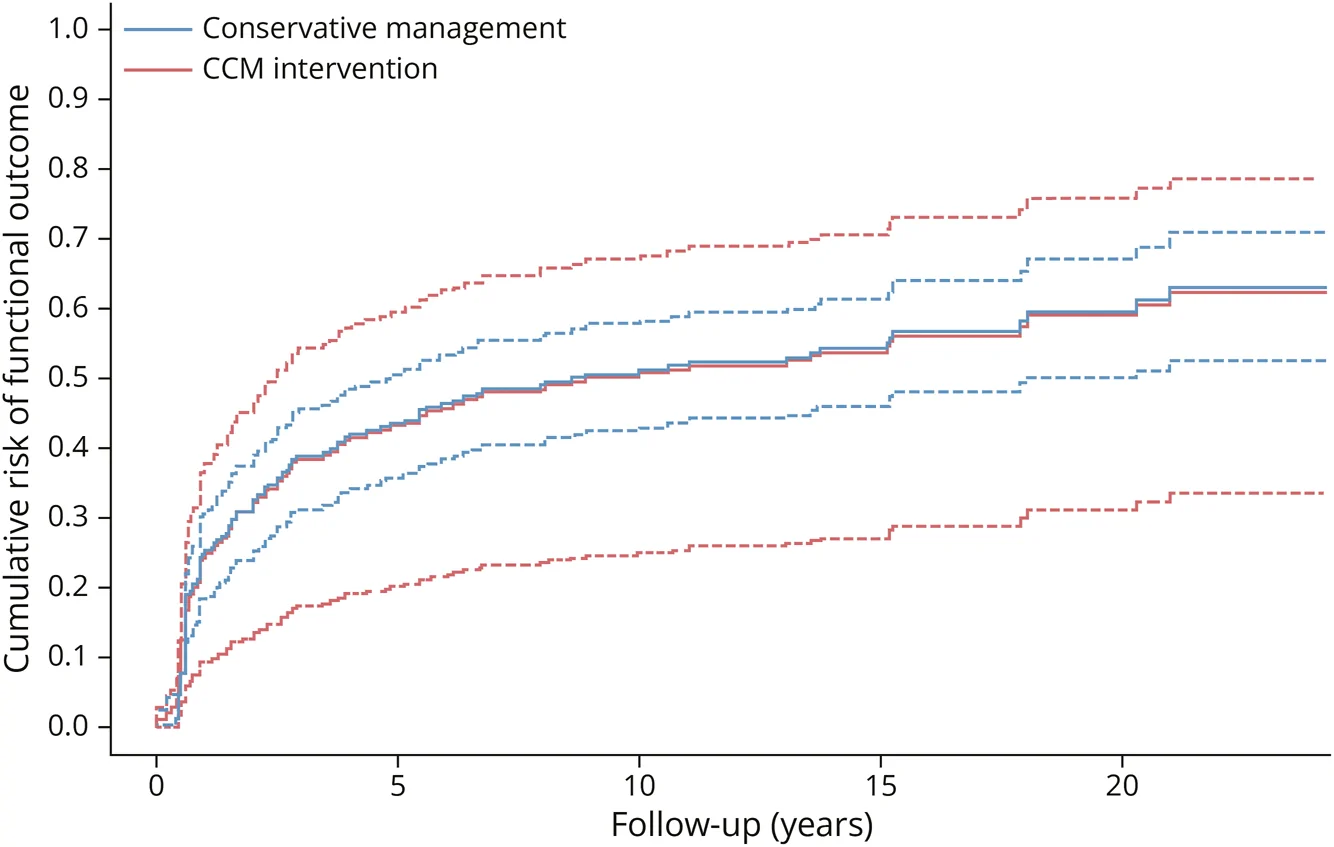
Cox Model–Based Estimated Cumulative Risk of Persistent Progression to OHS Scores of ≥2 for at Least 2 Consecutive Annual Ratings During Follow-Up by Treatment Status Dashed lines represent the 95% CI. CCM = cerebral cavernous malformation; OHS = Oxford Handicap Scale.

The primary outcome of persistent progression to OHS scores of ≥2 for at least 2 consecutive annual ratings during follow-up was experienced by 109 (57%) patients (n = 96 managed conservatively and n = 13 CCM intervention). CCM intervention was not associated in both univariable analysis (Figure 1) and multivariable analysis (adjusted HR 1.25 [95% CI 0.65–2.38], *p* = 0.50, Table 2). The risk was, however, increased in patients who were older, had higher deprivation scores, had worse inception OHS scores, experienced epileptic seizure(s) at inception, or had brainstem CCMs. In sensitivity analysis of OHS scores of ≥3 (n = 55 [29%], n = 44 managed conservatively, and n = 11 CCM intervention), CCM intervention was associated with an increased risk (adjusted HR 2.66 [95% CI 1.19–5.94], *p* = 0.017, eTable 4). Compared with the primary analysis, in addition to older age, higher deprivation scores, and worse inception OHS scores, we further found higher CCI scores to be associated, but not mode of presentation or presence of brainstem CCM.

**Table 2:** Cox Proportional Hazards Regression Analysis for Associations With the Occurrence of Persistent Progression to OHS Scores of ≥2 for at Least 2 Consecutive Annual Ratings During Follow-Up

Covariate	Unadjusted HR (95% CI)	*p* Value	Adjusted HR (95% CI)	*p* Value
CCM intervention (vs conservative management)	0.99 (0.53–1.85)	0.97	1.25 (0.65–2.38)	0.50
Age (10-y increments)	1.16 (1.03–1.30)	**0.013**	1.20 (1.05–1.38)	**0.010**
Female (vs male) sex	0.77 (0.53–1.11)	0.16	0.84 (0.56–1.27)	0.40
CCI score (1-unit increment)	1.14 (0.99–1.31)	0.07	1.05 (0.92–1.20)	0.49
Deprivation category (1-unit increment)	1.11 (0.99–1.25)	0.09	1.13 (1.00–1.28)	**0.047**
OHS score at inception (1-unit increment)	2.12 (1.56–2.88)	**<0.001**	2.35 (1.75–3.18)	**<0.001**
ICH/FND (vs epileptic seizure[s]) at inception	0.89 (0.61–1.31)	0.56	0.53 (0.33–0.87)	**0.011**
Presence (vs absence) of brainstem CCM	1.63 (1.01–2.64)	**0.045**	2.53 (1.42–4.49)	**0.002**
Multiple (vs single) CCMs	1.38 (0.88–2.17)	0.17	1.25 (0.77–2.00)	0.37
Largest CCM diameter (1-mm increments)	0.99 (0.96–1.01)	0.31	1.00 (0.97–1.03)	0.99

Abbreviations: CCI = Charlson Comorbidity Index; CCM = cerebral cavernous malformation; HR = hazard ratio; ICH/FND = symptomatic intracranial hemorrhage or new nonhemorrhagic persistent/progressive focal neurologic deficit; OHS = Oxford Handicap Scale.

Data are represented as HR (95% CI); *p *values presented in bold were considered statistically significant (*p* < 0.05).

The reciprocal outcome of sustained progression to OHS scores 0–1 for at least 2 consecutive annual ratings during follow-up was experienced by 156 (82%) patients (n = 135 managed conservatively and n = 21 CCM intervention). CCM intervention was not associated with this outcome (eTable 5). In the reciprocal sensitivity analysis of OHS scores 0–2 (n = 175 [92%], n = 159 managed conservatively, and n = 16 CCM intervention), CCM intervention was associated with a decreased rate (adjusted HR 0.59 [95% CI 0.37–0.95], *p* = 0.030, eTable 6).

### Clinical Outcomes

**Figure 2:**
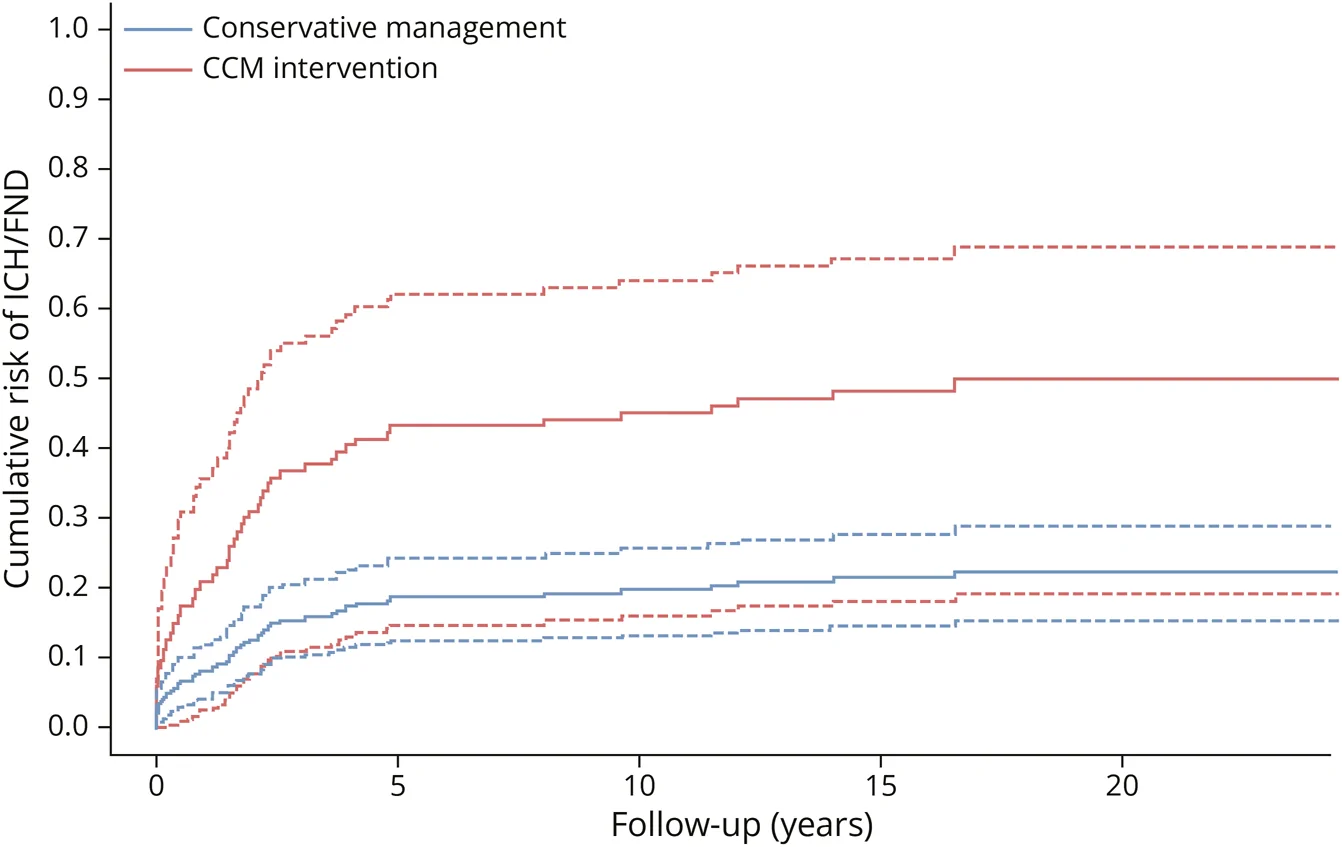
Cox Model–Based Estimated Cumulative Risk of ICH/FND Definitely or Possibly Related to CCMs or Attributable to CCM Intervention During Follow-Up by Treatment Status Dashed lines represent the 95% CI. CCM = cerebral cavernous malformation; ICH/FND = symptomatic intracranial hemorrhage or new nonhemorrhagic persistent/progressive focal neurologic deficit.

ICH/FND during follow-up was experienced by 45 (24%) patients (n = 35 managed conservatively, n = 10 CCM intervention, n = 29 definitely related to CCM, n = 8 possibly related to CCM, and n = 8 attributable to CCM intervention). CCM intervention was associated with an increased risk in univariable analysis (Figure 2), but not when adjusted for confounders (adjusted HR 2.81 [95% CI 0.94–8.42], *p* = 0.07, Table 3). The risk was, however, higher in patients with ICH/FND at inception (adjusted HR 3.26 [95% CI 1.37–7.77], *p* = 0.008) or brainstem CCM (adjusted HR 4.16 [95% CI 1.98–8.73], *p* < 0.001). In sensitivity analysis of ICH/FND that were only definitely related to CCM or attributable to CCM intervention (n = 37 [19%], n = 28 managed conservatively, and n = 9 CCM intervention), CCM intervention was associated with an increased risk (adjusted HR 3.59 [95% CI 1.05–12.20], *p* = 0.041) as well as ICH/FND at inception and presence of brainstem CCM (eTable 7).

**Table 3:** Cox Proportional Hazards Regression Analysis for Associations With the Occurrence of ICH/FND Definitely or Possibly Related to CCM or Attributable to CCM Intervention During Follow-Up

Covariate	Unadjusted HR (95% CI)	*p* Value	Adjusted HR (95% CI)	*p* Value
CCM intervention (vs conservative management)	2.75 (1.22–6.20)	**0.015**	2.81 (0.94–8.42)	0.07
Age (10-y increments)	1.06 (0.89–1.26)	0.55	0.92 (0.74–1.15)	0.48
Female (vs male) sex	1.49 (0.82–2.71)	0.19	0.94 (0.48–1.87)	0.87
ICH/FND (vs epileptic seizure[s]) at inception	5.13 (2.59–10.17)	**<0.001**	3.26 (1.37–7.77)	**0.008**
Presence (vs absence) of brainstem CCM	5.00 (2.78–9.00)	**<0.001**	4.16 (1.98–8.73)	**<0.001**
Antithrombotic medication at inception	1.13 (0.38–3.40)	0.83	1.16 (0.36–3.81)	0.80

Abbreviations: CCM = cerebral cavernous malformation; HR = hazard ratio; ICH/FND = symptomatic intracranial hemorrhage or new nonhemorrhagic persistent/progressive focal neurologic deficit.

Data are represented as HR (95% CI); *p *values presented in bold were considered statistically significant (*p* < 0.05).

For symptomatic ICH alone (excluding events of new nonhemorrhagic FND), which occurred in 21 (11%) patients (n = 19 managed conservatively, n = 2 CCM intervention, n = 19 definitely related to CCM, n = 1 possibly related to CCM, and n = 1 attributable to CCM intervention), CCM intervention was not associated, whereas symptomatic ICH at inception and presence of brainstem CCM were associated (eTables 8 and 9).

**Figure 3:**
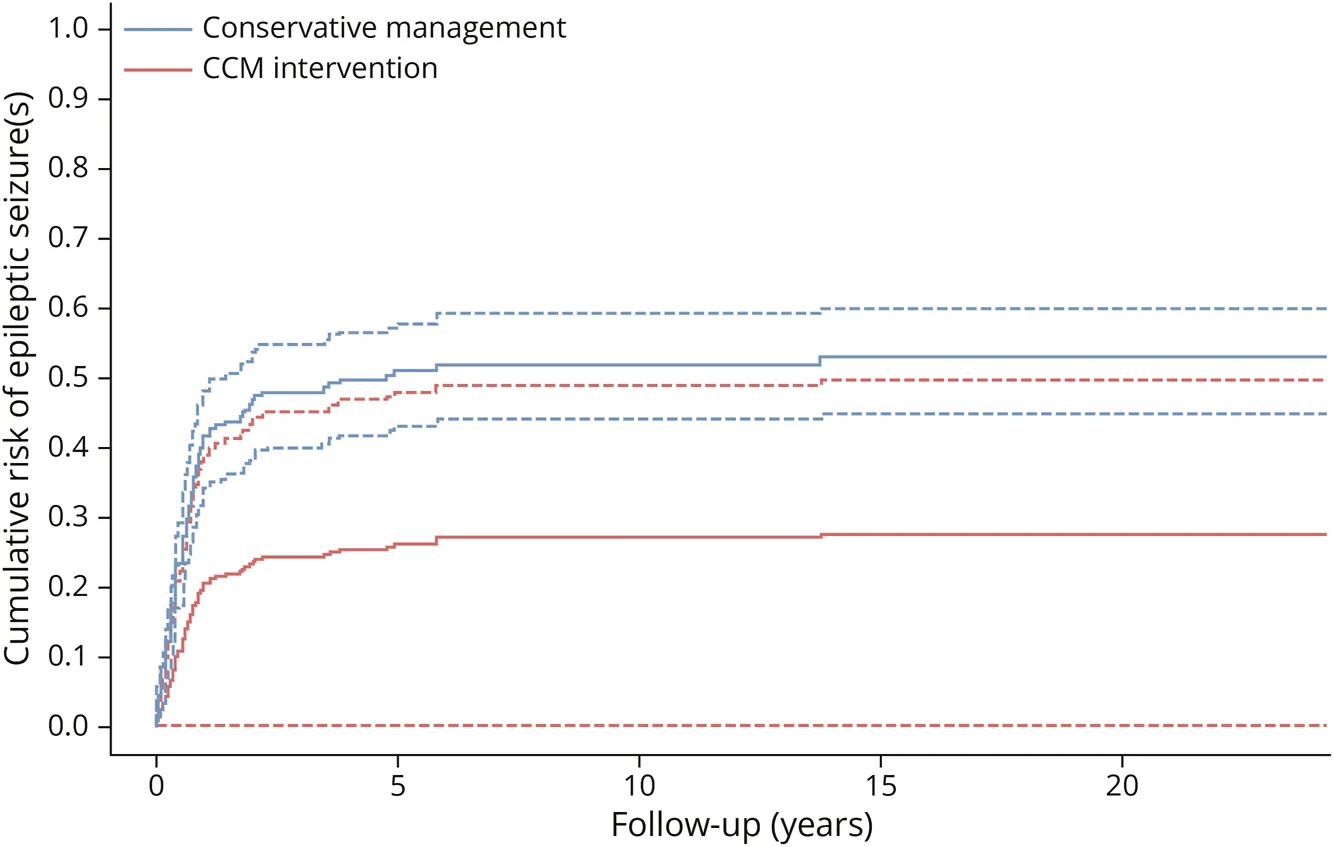
Cox Model–Based Estimated Cumulative Risk of Epileptic Seizure(s) Definitely or Possibly Related to CCMs During Follow-Up by Treatment Status Dashed lines represent the 95% CI. CCM = cerebral cavernous malformation.

Epileptic seizure(s) during follow-up were experienced by 95 (50%) patients (n = 92 managed conservatively and n = 3 CCM intervention). All events were definitely or possibly related to CCMs, and none were attributable to CCM intervention. CCM intervention was not associated in both univariable analysis (Figure 3) and multivariable analysis (adjusted HR 1.56 [95% CI 0.49–4.99], *p* = 0.45, Table 4). Adjusted for confounders such as antiseizure medication after inception (which was highly associated, adjusted HR 32.00 [95% CI 7.69–133.19], *p* < 0.001), the risk of epileptic seizure(s) decreased with age (adjusted HR 0.81 [95% CI 0.69–0.94] per 10-year increment, *p* = 0.006). In sensitivity analysis of epileptic seizure(s) that were only definitely related to CCMs (n = 86 [45%], n = 83 managed conservatively, and n = 3 CCM intervention), similar results were found, although it further showed that having multiple CCMs was associated with an increased risk (adjusted HR 1.59 [95% CI 1.01–2.51], *p* = 0.043, eTable 10).

**Table 4:** Cox Proportional Hazards Regression Analysis for Associations With the Occurrence of Epileptic Seizure(s) Definitely or Possibly Related to CCMs During Follow-Up

Covariate	Unadjusted HR (95% CI)	*p* Value	Adjusted HR (95% CI)	*p* Value
CCM intervention (vs conservative management)	0.42 (0.13–1.37)	0.15	1.56 (0.49–4.99)	0.45
Age (10-y increments)	0.71 (0.61–0.82)	**<0.001**	0.81 (0.69–0.94)	**0.006**
Female (vs male) sex	0.54 (0.36–0.82)	**0.003**	0.89 (0.58–1.34)	0.57
Epileptic seizure(s) (vs ICH/FND) at inception	12.92 (6.72–24.82)	**<0.001**	1.41 (0.58–3.42)	0.44
Presence (vs absence) of temporal cortical/superficial CCM	2.80 (1.90–4.12)	**<0.001**	1.02 (0.67–1.54)	0.93
Antiseizure medication after inception	43.40 (15.84–118.90)	**<0.001**	32.00 (7.69–133.19)	**<0.001**
Multiple (vs single) CCM	1.38 (0.87–2.17)	0.17	1.38 (0.94–2.03)	0.10
Largest CCM diameter (1-mm increments)	1.00 (0.97–1.03)	0.88	0.99 (0.96–1.02)	0.50

Abbreviations: CCM = cerebral cavernous malformation; HR = hazard ratio; ICH/FND = symptomatic intracranial hemorrhage or new nonhemorrhagic persistent/progressive focal neurologic deficit.

Data are represented as HR (95% CI); *p *values presented in bold were considered statistically significant (*p* < 0.05).

## Discussion

In this prospective, population-based cohort study of symptomatic CCM, we did not find an association between CCM intervention and progression to at least 2 consecutive annual OHS score ratings of ≥2 or 0–1. However, in sensitivity analysis of OHS scores of ≥3 or 0–2, CCM intervention was associated with higher risk of persistent functional dependence or death and a lower reciprocal rate of sustained independence, respectively. Similarly, in primary analysis of ICH/FND definitely or possibly related to CCM or CCM intervention during follow-up, we found no association, but in sensitivity analysis without possibly related events, CCM intervention was associated with an increased risk. CCM intervention was not associated with higher or lower risk of epileptic seizure(s) during follow-up.

One of the most pressing questions in CCM clinical practice and research is whether patients diagnosed with symptomatic CCM should either undergo CCM intervention or be managed conservatively. Unlike indirect comparisons using historical or synthetic controls, comparative studies with concurrent control groups provide higher certainty evidence by minimizing confounding due to differences in baseline risks and secular trends, and ensuring consistent outcome measurement. A few direct comparative studies have been published,^9^ including a systematic review.^22^ However, none demonstrated a substantial benefit of CCM intervention over conservative management in patients with symptomatic CCMs. Moreover, 2 systematic reviews with meta-analysis reported conflicting results^23,24^ because one suggested that CCM intervention was superior for preventing neurologic deficits,^23^ whereas the other concluded that conservative management was associated with a longer time to focal neurologic deficit.^24^

A systematic review and meta-analysis of 100 cohort studies, many of which evaluated either 1 type of CCM intervention or conservative management, suggested that the outcomes were more favorable after microsurgical resection.^25^ However, the conclusion was based on cumulative incidence estimates derived from mean follow-up periods of less than 5 years, while untreated risks of recurrent events from CCM decline over time,^6,7^ with substantially lower risks beyond 5 years,^6^ and the approach did not account for the long-term outcomes after CCM intervention. Furthermore, treatment modalities were compared across separate cohorts without adjustment for confounding variables, such as the lower mean age of patients treated with surgery. The extent of additional confounding could not be assessed because only absolute patient numbers were reported for key characteristics (e.g., brainstem location, multiple CCMs, and presentation with ICH). Notably, some of the included studies exclusively reported on brainstem CCMs, a location consistently associated with an increased risk of hemorrhage and disability.^26^ These methodological limitations, together with the conflicting findings across several reviews, underscore the need for robust comparative evidence in patients with symptomatic CCM.

All the aforementioned reviews included our initial cohort of patients with CCM diagnosed between 1999 and 2003 in Scotland,^10^ which is also included in this study. However, the previous analysis compared patients who underwent CCM intervention at any time during follow-up with those managed conservatively throughout. This may have introduced an immortal time bias because patients classified in the intervention group were required to survive and remain free of events that would preclude intervention (e.g., severe ICH, neurologic deterioration, or death). Consequently, a period of guaranteed event-free survival may have been attributed to the intervention group, potentially underestimating the true risk associated with CCM intervention. In the current study, we addressed this limitation by modelling CCM intervention as a time-dependent covariate. In addition, the extended follow-up provides estimates of long-term outcomes.

We found that CCM intervention was associated with an increased risk of ICH/FND during follow-up in sensitivity analysis of only events definitely related to CCMs, whereas in the initial cohort, an increased risk was also observed in the primary analysis.^10^ One explanation is that the earlier study included both incidental and symptomatic cases, with incidental CCMs predominantly in the conservative group. In this study, we restricted our analysis to patients with symptomatic CCMs because clinical equipoise exists primarily in this group and only these patients would be eligible for inclusion in an RCT.^4,27^ Moreover, inclusion of incidental cases alters the clinical research question, shifting the focus toward prophylactic intervention generally driven by patient preference rather than preventing recurrent symptoms or progression of disability. Furthermore, it would lead to different time zero definitions. We defined inception as the date of the first symptomatic event, whereas for incidental CCM, the appropriate start of analysis would be less clear because of the uncertain timing of the development of CCM relative to diagnosis.

Although CCM intervention may prevent recurrent ICH/FND or epileptic seizure(s), the procedure itself carries a risk of ICH/FND and treatment-related disability. Overall, we did not find convincing evidence that CCM intervention was associated with ICH/FND or epileptic seizure(s) during follow-up, although a higher risk of ICH/FND was observed in sensitivity analysis. We did find a presentation with seizures—now recognized as CCM-related epilepsy (CRE)^7^—to be independently associated with a higher risk of functional impairment or death. For patients diagnosed with CRE, surgery was the preferred strategy in a decision analysis.^28^ However, CCM intervention is rarely performed after only a single seizure because patients often achieve seizure freedom with medical therapy alone.^7^ Therefore, evaluations of surgical benefit may be more appropriately focused on patients with medically refractory CRE, ideally using an RCT.

Neurologic impairments after CCM intervention usually occur in the short term, which motivated our requirement for at least 2 consecutive OHS score assessments to define persistent impairment. The differing results between OHS thresholds ≥2 and ≥3 may reflect that scores of ≥2 capture relatively mild impairments that improve, whereas more persistent impairments are more likely to occur after CCM intervention, although the OHS is patient-specific rather than disease-specific and comorbidities may also have contributed. Among patients who underwent CCM intervention, none of the 8 individuals with early procedural complications experienced recurrent clinical events thereafter, and only 2 individuals without early complications developed ICH/FND during long-term follow-up. Nevertheless, the group-level risk of persistent functional dependence or death may primarily reflect the impact of early complications, such as ICH/FND, attributable to CCM intervention. As compared with the publication of the initial cohort,^10^ this association persisted during extended follow-up, despite the possibility of capturing new events in patients managed conservatively, but this new event rate may be negligible because untreated risks of events decline over time.^6,7^

We additionally analyzed favorable functional outcome. The high number of such outcome events suggests favorable functional status at baseline, consistent with the inception OHS scores observed in our cohort and prior evidence showing that clinical manifestations of CCM are usually less severe than that of other intracranial (predominantly arterial) vascular malformations.^15^ Alternatively, it may reflect substantial spontaneous recovery after a symptomatic presentation. Occurrence of many events early after inception indicates early recovery that may precede CCM intervention, limiting exposure times and the relevance of a time-dependent covariate. Moreover, while favorable functional outcomes in the intervention group may reflect treatment benefit or lack of treatment-related harm, it might reflect the benign natural history with conservative management. We therefore selected persistent functional impairment or death as primary outcome, capturing patients who fail to achieve durable recovery or progress to lasting disability.

Strengths of this study include its comprehensive case ascertainment, independent imaging review and outcome assessment, and application of robust statistical methods to evaluate treatment of symptomatic CCMs in a population-based cohort with prospective long-term follow-up, thereby reducing selection, information, and immortal time biases. The principal limitation is the nonrandomized assessment of treatment strategies. Although we controlled for measured baseline imbalances that could confound the relationship between CCM intervention and outcomes, this study only estimates causal effects, lacking definitive causal inference. Additional approaches, such as propensity score matching, have been considered, but given that only 37 patients underwent CCM intervention, matching would likely have yielded few pairs, as observed in a previous study.^9^ Moreover, microsurgical resection and SRS were analyzed in a composite exposure. Although this may introduce false parity between treatment modalities, its effect is likely minimal because only 2 cases underwent SRS. In addition, potential changes over time in patient selection for CCM intervention, advances in treatment techniques, and differences in surgeons' or centers' experience may represent sources of residual confounding that could not be adjusted for. We were also unable to adjust for time-varying clinical events that both influence the likelihood of intervention and affect functional outcome when the event and procedure occurred between 2 OHS score assessments. Furthermore, we included all cases of symptomatic CCM, whereas a target trial would likely exclude patients with clear indications or contraindications for intervention. Such distinctions could not be reliably identified in our study. Another limitation is that estimates for the outcome of persistent functional impairment (defined as OHS scores ≥3) were relatively imprecise because of the small number of such events. Finally, seizure outcomes should be interpreted with caution because details on factors such as resection of the hemosiderin rim, the type, number, dosage, and adherence of antiseizure medication, and standardized outcome ascertainment (e.g., using electroencephalography) were lacking.

This observational study provides comparative evidence on the management of symptomatic CCMs in the absence of a definitive RCT. CCM intervention was associated with a higher risk of persistent functional dependence or death and a lower rate of sustained functional independence, findings that may largely reflect early procedure-related complications, such as ICH/FND. We did not find evidence that CCM intervention was associated with epileptic seizure(s) during follow-up, although CCM intervention is rarely performed after the first seizure. To determine whether CCM intervention or conservative management is superior for patients with symptomatic CCM, a definitive RCT with long-term follow-up is needed.^5^ Although a recent pilot phase RCT demonstrated the feasibility of such a trial, it indicated that large-scale international, multicenter collaboration would be required,^5^ highlighting the logistical challenges of studying a rare condition such as symptomatic CCM.^29^ A platform trial might represent a cost-effective alternative, allowing evaluation across different modes of presentation (e.g., ICH/FND or CRE) while incorporating key prognostic factors such as brainstem location.^30^ Pending such evidence, the findings from this study may assist clinicians and patients with symptomatic CCMs in shared decision-making regarding intervention or conservative management.

## Glossary

CCI: Charlson Comorbidity Index
CCM: cerebral cavernous malformation
CRE: CCM-related epilepsy
FND: focal neurologic deficits
HR: hazard ratio
ICH: intracranial hemorrhage
IQR: interquartile range
OHS: Oxford Handicap Scale
RCT: randomized controlled trial
SAIVMs: Scottish Audit of Intracranial Vascular Malformation
SRS: stereotactic radiosurgery
